# Relationship between Ultrasound Backscattered Statistics and the Concentration of Fatty Droplets in Livers: An Animal Study

**DOI:** 10.1371/journal.pone.0063543

**Published:** 2013-05-21

**Authors:** Ming-Chih Ho, Yu-Hsin Lee, Yung-Ming Jeng, Chiung-Nien Chen, King-Jen Chang, Po-Hsiang Tsui

**Affiliations:** 1 Department of Surgery, National Taiwan University Hospital and College of Medicine, National Taiwan University, Taipei, Taiwan; 2 Angiogenesis Research Center, National Taiwan University, Taipei, Taiwan; 3 Department of Pathology, National Taiwan University Hospital and College of Medicine, National Taiwan University, Taipei, Taiwan; 4 Department of Medical Imaging and Radiological Sciences, College of Medicine, Chang Gung University, Taoyuan, Taiwan; 5 Healthy Aging Research Center, Chang Gung University, Taoyuan, Taiwan; Wageningen University, The Netherlands

## Abstract

Ultrasound grayscale B-mode imaging is the most frequently used modality for examining fatty liver. Different concentrations and arrangements of fatty droplets in the liver may produce different statistical distributions of ultrasound backscatter signals, which may be treated as a useful clue for assessing the stage of fatty liver. To verify this point, we investigate the relationship between changes in backscattered statistics and the concentration of fatty droplets in the liver. Fatty liver was induced in rats fed a methionine-choline-deficient diet. Livers were excised from rats for in *vitro* ultrasound scanning using a single-element transducer. The envelopes of the acquired raw ultrasound signals were used for the analysis of the backscattered statistics by ultrasound Nakagami parametric imaging, which has been shown as a reliable tool to model the statistical distribution of ultrasound backscatter signals. Histological analyses and the measurements of triglyceride and cholesterol in the rat liver were conducted for comparison with the Nakagami parameter. Results show that the ultrasound Nakagami parameter has an excellent correlation with the concentration of fatty droplets, demonstrating that ultrasound backscatter statistics depend on the degree of fatty liver in rats.

## Introduction

Fatty liver is a reversible condition in which large vacuoles of triglyceride fat accumulate in liver cells through the process of steatosis (i.e., an abnormal retention of lipids within a cell). It is the most common cause of abnormal liver function in patients without viral hepatitis. The most common causes of fatty liver are alcoholism, obesity, insulin-resistant diabetes mellitus, and hyperlipidemia [1]. The presence of fatty liver may be treated as a “marker” of many diseases [2]. Nonalcoholic fatty liver was reported to be a risk factor for postprandial hyperglycemia [3]. This condition is also closely associated with hypertriglyceridemia and hyperuricemia, even in non-obese, non-diabetic subjects. Changes in the degree of fatty liver may reflect the response to treatments. The daily physical activity attenuated hepatic steatosis in an obese rodent model [4]. Steatosis or steatohepatitis may also improve or resolve significantly in 80% of obese patients receiving bariatric surgery [5]. The detection of changes in fatty liver and the precise evaluation of the severity are critical to the prevention and treatment of fatty liver-associated comorbidity.

Liver biopsy is the gold standard for fatty liver diagnosis and evaluation. Histological examination provides the percentage of fat accumulation and tissue changes to the cellular level. However, this method is not frequently used in clinical practice because of its invasive nature. The risks of infection, bleeding, and bile leak, although not frequent, do exist [6]. This is the reason why noninvasive imaging techniques are frequently used to detect and quantify the severity of fatty liver in general practice.

Computed tomography (CT) is a useful noninvasive tool to quantify hepatic steatosis [7], [8]. The spleen-to-liver attenuation ratio, or the difference between the attenuation of the spleen and liver, has been proposed to evaluate hepatic steatosis [9], [10]. Some reports have introduced the measurement of the same image with 2 different kVp values [11], [12]. This approach can achieve good CT performance for the diagnosis of significant steatosis in live liver donors [13], [14]. Magnetic resonance imaging (MRI) has also shown promise in the evaluation of hepatic steatosis using the breath-hold T1-weighted gradient-echo in–/out-of-phase sequence [15], [16]. However, in patients with coexisting iron deposition and other parenchymal diseases, the diagnostic performance of CT for the quantitative assessment of macrovesicular steatosis may not be clinically acceptable. Moreover, the CT and MRI examinations are not available in all circumstances. Special equipment and space are also needed, and image acquisition, calculation, and analysis take time. In addition, radiation exposure, and occasionally, contrast medium are unavoidable. These examinations are impractical for fatty liver screening or evaluating the general population.

Ultrasound is the most frequently used modality for examining liver disease because of its convenience, real-time, high resolution, low cost, and lack of radiation exposure. Generally, liver echogenicity increases with the severity of steatosis. This allows the semiquantitative evaluation of the degree of hepatic steatosis. However, the ultrasonographic evaluation of steatosis is subjective [17] and operator-dependent. Many system factors (e.g., gain, time gain compensation, and filtering) are able to affect image brightness, contrast, and resolution. Ultrasound can neither precisely reflect the histopathological quantification of steatosis, nor can it detect small changes in liver fat using grayscale images. These drawbacks limit the use of ultrasound in longitudinal studies. To date, grayscale B-mode ultrasound is not considered a replacement for liver biopsy in evaluating fatty liver [18].

In the B-mode image of medical ultrasound, the system usually removes noise caused by ultrasound backscattering to achieve a better image quality. However, this process removes biological information contained in the noise. The interactions between the incident ultrasound wave and the acoustical scatterers in a tissue produce the ultrasound backscattering. Therefore, the behavior of ultrasound backscattering may depend on size, shape, concentration, and other properties of the scatterers in the tissue. In other words, the backscattered signals returned from tissues may contain very useful information associated with the scatterer properties of a tissue. Based on the randomness of the backscattered echoes, the probability distribution of the backscattered echoes can be described using some mathematic statistical models for data analysis and tissue characterization.

In this study, we hypothesize that different concentrations and arrangements of scatterers (i.e., the fatty droplets in a liver tissue) correspond to different statistical distributions of the backscattered signals. Based on this concept, the analysis of the backscattered statistics of ultrasound image may provide useful clues for fatty liver diagnosis. To validate the proposed viewpoint, we explored the relationship between the backscattered statistics and the concentration of fatty droplets in the liver by rat model.

## Materials and Methods

### Animal Model

The institutional animal care and use committee in Taiwan University Hospital approved the use of the rats in this study. Twenty-four male Wistar rats weighing 210–250 g that were 7 weeks of age were used in this study. Prior to the experiments (day 0), 3 rats were firstly sacrificed to excise left lateral lobes of the livers as the samples of the control group. The other rats were housed in a temperature-controlled room (22±1°C) with a relative humidity of 50±10% and a 12 h light/dark cycle. Rats were fed with a high fat/methionine-choline-deficient diet (MCD; Baker Amino Acid Diet lacking choline and methionine 578 B; TestDiet, Richmond, IN) ad libitum for 7 days to induce fatty livers. In each day, 3 rats were chosen in random to be sacrificed. Liver samples excised from rats were used for in *vitro* ultrasound scanning and histological examinations.

### *In vitro* Ultrasound Scanning

**Figure 1:**
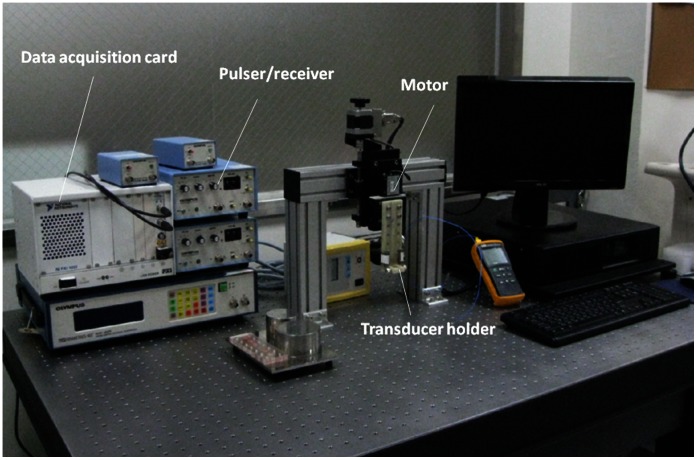
The experimental setup for ultrasound scanning of rat livers in vitro.

A single-crystal ultrasound imaging system was constructed for scanning the liver in *vitro* to acquire the ultrasonic backscattered signals associated with different degrees of fatty liver (Fig. 1). The system included a mechanical scanning assembly, a single-element transducer, a pulser/receiver, and a data acquisition card. The transducer was mechanically scanned using a high-resolution motion stage driven by a piezoelectric motor (Model HR8, Nanomotion, Yokneam, Israel). The transducer was driven by a pulser/receiver (Model 5072 PR, Panametrics-NDT, Waltham, MA, USA) for transmitting and receiving ultrasonic signals. The received RF echoes that were backscattered from the liver were amplified with the built-in 59 dB amplifier in the pulse/receiver and then digitized using an analog-to-digital converter (Model PXI-5152, National Instruments, Austin, TX, USA) for data storage and offline analysis on a personal computer.

The focused transducer used in this study had an element diameter of 6 mm (Model V310, Panametrics-NDT). The pulse-echo test of the transducer showed that the central frequency and pulse length were 6.5 MHz and 0.3 mm, respectively. The focal length was 1.1 cm, and therefore, the theoretical -6 dB beam width calculated using the f-number was approximately 0.4 mm. The transducer and liver samples were placed on a holder in a bath containing saline solution at room temperature. The liver was positioned at the focus of the transducer (1.1 cm away). Five independent B-scans were performed for each liver specimen. Each B-scan consisted of 100 A-lines of the backscattered signals obtained at a sampling rate of 50 MHz. Each backscattered signal corresponded to a data length of approximately 12 mm. The interval between each A-line was 0.1 mm. Each scan line was then demodulated using Hilbert transform to obtain the envelope image, and the B-mode image was formed based on the logarithm-compressed envelope image at a dynamic range of 40 dB.

### Backscattered Statistics Analysis

**Figure 2:**
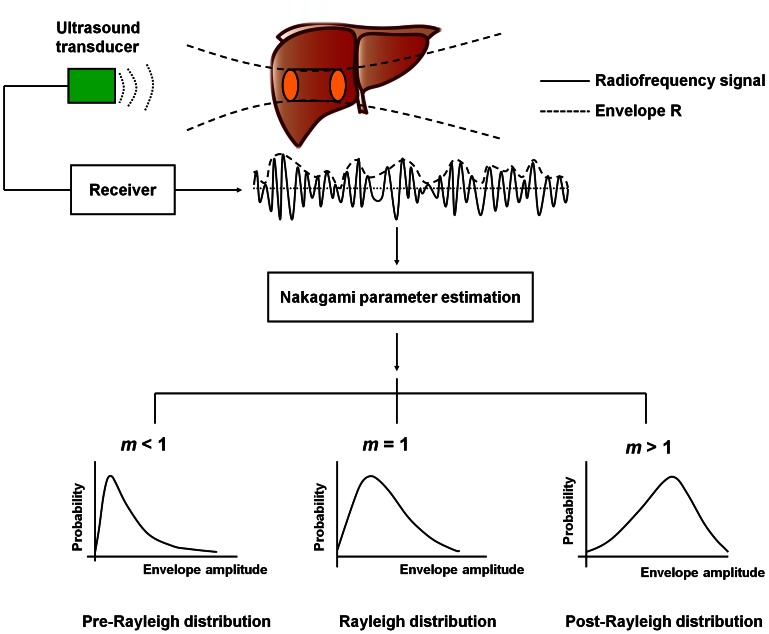
Raw ultrasound backscattered signals and the corresponding envelope signal can be used to estimate Nakagami parameter *m* to model the statistical distribution of backscattered-signal envelope *R*. Different Nakagami parameters indicate different envelope statistics caused by different properties of scatterers in the tissue.

This study used ultrasound Nakagami imaging based on a 2 D Nakagami parameter map to analyze the statistical distribution of ultrasound signals. Nakagami imaging produces reliable images to reflect backscattered statistics [19], [20], [21], [22]. The Nakagami parameter is a shape parameter of the Nakagami statistical distribution, as given by [23]

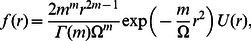
(1)where Γ(•) and U(•) are the gamma function and the unit step function, respectively. Let E(•) denote the statistical mean. The scaling parameter 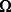 and the Nakagami parameter m associated with the Nakagami distribution can then be respectively obtained from
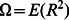
(2)and



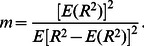
(3)The variation of the Nakagami parameter from 0 to 1 indicates a change in the envelope statistics from pre-Rayleigh to Rayleigh distribution, and a Nakagami parameter larger than 1 means that the backscattered statistics conform to post-Rayleigh distributions. The statistics of the backscattered envelope follows the Rayleigh distribution when the resolution cell of the ultrasonic transducer contains a large number of randomly distributed scatterers. If the resolution cell contains scatterers that have randomly varying scattering cross-sections with a comparatively high degree of variance, the envelope statistics are pre-Rayleigh distributions. If the resolution cell contains periodically located scatterers in addition to randomly distributed scatterers, the envelope statistics are post-Rayleigh distributions, as shown in Fig. 2. Because the Nakagami distribution with a distribution shape determined by the Nakagami parameter fits well with the envelope histogram of the ultrasonic backscatter signals [20], the Nakagami distribution is a general model for ultrasonic backscattering.

Previous studies have presented the details of Nakagami imaging [19]. The Nakagami image is based on the Nakagami parameter map, which is constructed using a local sliding window to process the raw envelope image. This process involves two main steps:

- A window within the envelope image collects the local backscattered envelopes for estimating the local Nakagami parameter m_w_, which is assigned as the new pixel located in the center of the window.
- Step (i) is repeated with the window moving throughout the entire envelope image in steps of one pixel. This step yields the Nakagami image as the map of m_w_ values.

Previous studies have suggested using a square with a side length equal to 3 times the pulse length of the incident ultrasound as a sliding window to construct the Nakagami image. This approach can simultaneously provide stable estimations of m_w_ and achieve acceptable imaging resolution [19]. This study constructs Nakagami images using a square window measuring 0.9×0.9 mm^2^.

### Histological Analysis

To evaluate the degree of fatty liver, excised liver tissues were embedded in OCT media (Tissue-Tek O.C.T compounds; Sakura Finetek, Torrance, CA) and slowly frozen at −80°C. For histological examination, 5-µm-thick sections were cut and then stained with Oil Red O.

### Lipid Concentration Estimation of the Liver

To determine lipid concentration, the liver tissue was homogenized with a 2∶1 chloroform-methanol mixture (v/v) to a final dilution 20× the volume of the tissue sample. The homogenate was filtered through filter paper into a glass tube. Triglyceride and cholesterol were extracted from liver samples and measured by enzymatic methods using commercial kits for triglyceride and cholesterol, respectively (Fortress Diagnostic).

### Data Comparisons and Statistical Analysis

Pearson correlation coefficients were used to assess the correlation among the Nakagami images, cholesterol, and triglyceride concentration in the liver tissue. This study presents all data as the mean ± standard deviation (SD). Statistical significance was defined as *p*<0.05.

## Results

**Figure 3:**
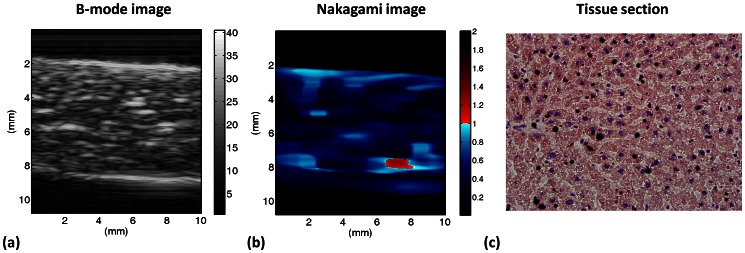
B-mode, Nakagami, and tissue section images (40× magnification) of a normal rat liver.

**Figure 4:**
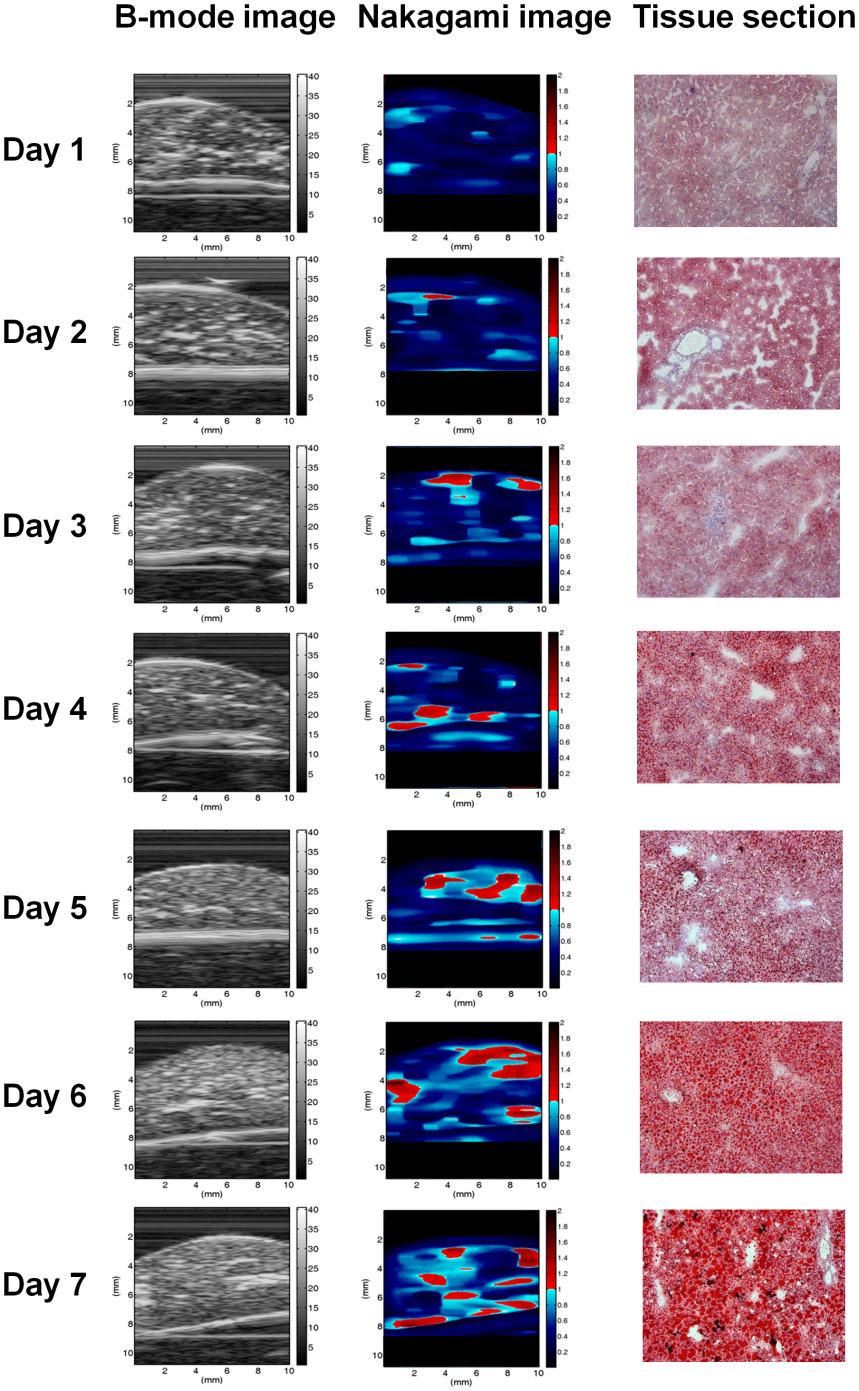
Tissue sections, B-mode, and Nakagami images of rat livers with different degrees of fatty liver.

Figure 3 shows the B-mode, Nakagami, and tissue section images of a normal rat liver. Figure 4 shows typical images of the tissue section for rat livers with various degrees of fatty liver and the corresponding B-mode and Nakagami images. The number of red shading pixels in the tissue sectional image gradually increased with an increase in MCD diet time, which successfully induced various degrees of fatty liver. The B-scan image seems to roughly reflect the progress of fatty liver in the rats because the image intensity increased slightly with MCD diet time. Note that the B-mode images have horizontal strip-shaped background lines, especially in the top of the images. The background signals were formed due to the damping signal, which is the small unwanted residual vibration of the transducer excitation pulse. These signals could produce undesirable shadings (i.e., artifact) in the Nakagami image. For this reason, we just selected the region of liver tissue for Nakagami imaging.

**Figure 5:**
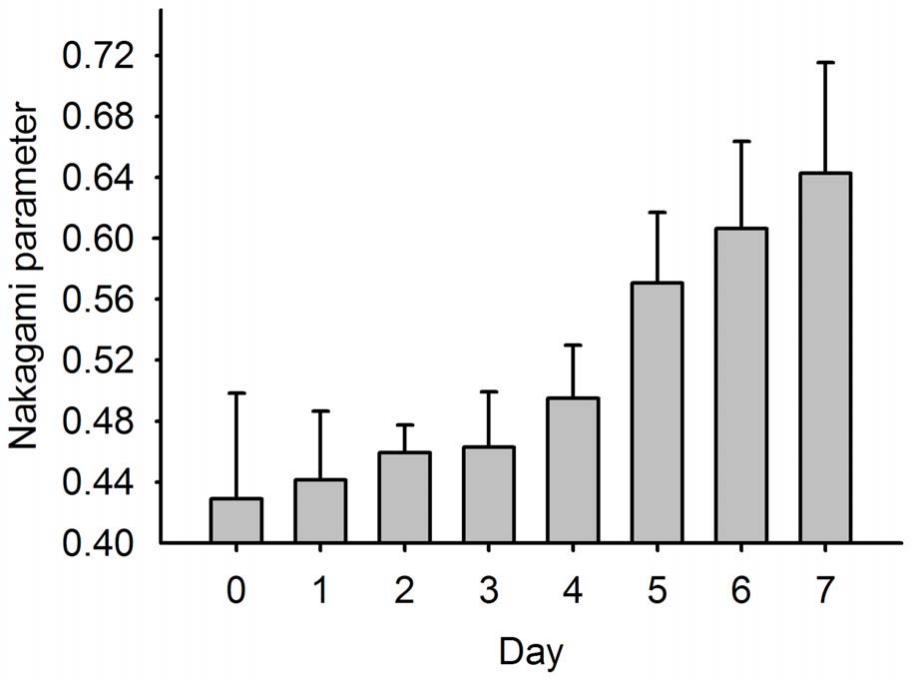
The Nakagami parameter as a function of MCD diet time.

We found that the information provided by Nakagami imaging regarding the degree of fatty liver differed from that available in the B-scan image. The shading of the Nakagami image is apparently correlated with the formation of fatty liver in rats because the amount of the red–blue-interlaced shading in the Nakagami images of the rat livers increased with the degree of fatty liver. This means that the backscattered statistics varied with the formation of fatty liver. Blue shading (i.e., pre-Rayleigh distribution) was predominant in the Nakagami image of a reference rat liver, whereas the amount of red shading (i.e., indicative of regions following post-Rayleigh distributions) increased with the fatty liver. In this condition, the average Nakagami parameter corresponds to a dynamic range of approximately 0.43 to 0.65, indicating that the global backscattered statistics vary toward the direction of the Rayleigh distribution during fatty liver formation, as shown in Fig. 5. The experimental results indicated that the statistics of the backscattered signals and the corresponding estimation of the Nakagami parameter are correlated with the degree of fatty liver.

**Figure 6:**
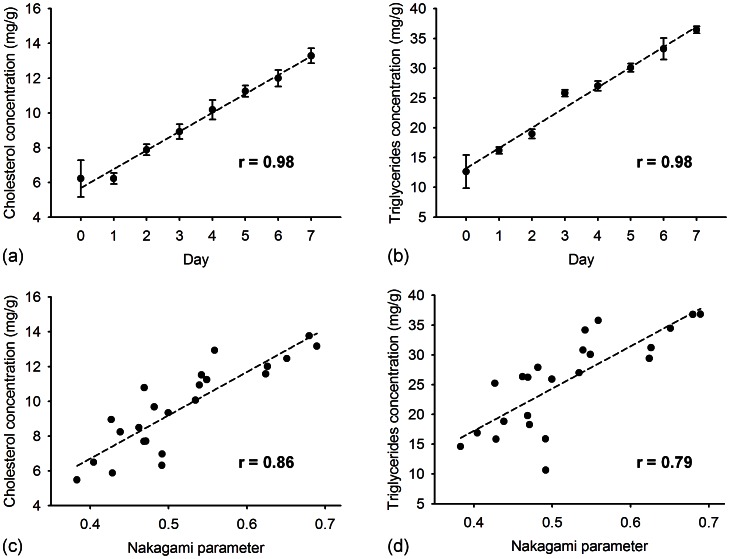
The concentrations of cholesterol and triglycerides in the liver as a function of MCD diet time are shown in (a) and (b), respectively (*p*<0.0001). The concentrations of cholesterol and triglycerides in the liver as a function of Nakagami parameter are shown in (c) and (d), respectively (*p*<0.0001).

The cholesterol and triglyceride concentrations of liver tissue were measured using the enzymatic procedure for each rat. Figures 6(a) and (b) show the concentrations of cholesterol and triglycerides in the rat liver as a function of the MCD diet time, respectively. This demonstrates that the cholesterol and triglyceride concentration increased as the MCD diet time increased (for both *r* >0.9; *p*<0.0001). Figures 6(c) and (d) show a comparison of the Nakagami parameter with the cholesterol and triglyceride results. These results indicate that the ultrasonic quantification of fatty liver using Nakagami parameters is well correlated with the amount of the total cholesterol (*r* = 0.86; *p*<0.0001) and triglyceride (*r* = 0.79; *p*<0.0001) in the liver tissue, respectively. The relationship between the Nakagami parameter and lipid concentration shows that different concentrations of fatty droplets lead to different backscattered statistics in the ultrasound signals.

## Discussion

In conventional grayscale imaging, echogenicity increases with fatty infiltration progress in the liver tissue, compared with the surrounding organs such as the spleen and kidney. Posterior attenuation becomes more significant as fatty liver severity increases. Grayscale ultrasound remains the most frequently used technique for diagnosing fatty liver. However, the increased echogenicity of the liver is difficult to standardize because it varies according to ultrasound users and operation experience. An objective and quantitative parameter is needed for longitudinal follow-up, one that monitors the treatment response of fatty liver patients.

In this study, ultrasound Nakagami imaging was proposed to analyze the distribution of ultrasound backscattered signals. The experimental results showed an excellent correlation between the Nakagami parameter and the degree of steatosis in the rat liver, demonstrating that the backscattered statistics depends on the severity of fatty change in the liver tissue. In a recent study, the statistical parameters of the homodyned K distribution were used to explore the relationship between the backscattered statistics and the fatty liver by carrying out measuring the liver samples extracted from rabbits in *vitro* [24]. The parameters of the homodyned K distribution were found to significantly increase with increasing the degree of fatty liver, representing that the envelope statistics may be useful in detecting and characterizing fatty liver diseases. Our results agree well with those obtained in the previous study [24]. However, it is worthy emphasizing that this paper we presented is the first study to provide the direct and solid evidences (the analysis of the components for triglyceride and cholesterol) to demonstrate the dependency of the backscattered statistics on the severity of fatty change in the liver tissue. The Nakagami parameter is not only correlated with the fatty ratio obtained from pathological images, but it also reflects the concentrations of triglyceride and cholesterol in the liver tissue. According to experimental findings, the Nakagami parameter and imaging have great potential in assessing the stage of fatty liver.

Exactly how the Nakagami image can be used to assess the fatty liver is still unclear, but this study provides possible explanations. The formation of fatty droplets in the liver involves changing the arrangement and concentration of the scatterers. Although the term “scatterer concentration” is a theoretical concept, it is still possible to indirectly estimate scatterer concentrations in tissues by analyzing the statistical distribution of backscattered envelopes [25]. This is because the ultrasonic backscattered statistics varies with the scatterer concentration, as verified by computer simulations and phantom experiments [26], [27]. For a homogeneous medium, it has been shown that the backscattered statistics vary from a pre-Rayleigh to Rayleigh distribution as the concentration of acoustic scatterers in a tissue increases [23], [26], [27]. In the early stage of fatty liver, the concentration of fatty droplets is relatively low, and thus, the statistical distribution of the backscattered envelope exhibits pre-Rayleigh statistics. For the late stage of fatty liver, these results indicate that the global backscattered statistics exhibit a Rayleigh distribution. This may be the result of a higher concentration of fatty droplets in the liver.

Prior to the proposal of using the analysis of the backscattered statistics as a new concept to measure the fatty liver, some acoustic parameters have also been proposed to explore the feasibilities in assessing the stage of fatty liver. In the previous studies, the acoustic parameters including ultrasound attenuation and backscatter coefficient, defined as the differential scattering cross-section per unit volume in the 180° direction, were explored [28], [29]. The previous studies have indicated that the mean backscatter coefficient with fatty liver infiltration was higher than that in healthy cases. The ultrasound attenuation of the patients with fatty livers is also higher than that for normal cases. The image texture analysis has also been explored in the detection of fatty liver [29]. Among all texture indices, co-occurrences sum entropy and co-occurrences entropy can present the best results in fatty liver measurement. In our opinion, the backscatter coefficient, attenuation, and texture analysis may not not the best choices for fatty liver measurement in practice. The calculation of the backscatter coefficient needs the compensation of ultrasound attenuation effect along the acoustic path in the liver and the backscatter coefficient of the reference phantom. The attenuation coefficient only provides an overall evaluation of the whole liver tissue and cannot describe local tissue properties. On the other hand, performing the texture analysis is typically based on the grayscale image data provided from the ultrasound system (not the raw data). Therefore, the performance of the texture analysis in characterizing tissues is also affected by the system factors. The above weaknesses hinder the practical applications in clinical situations.

Evaluating the degree of fatty liver by measuring the tissue stiffness is the recently attractive method. Ultrasound elastography based on acoustic radiation force impulse (ARFI) has been studied to investigate its clinical usefulness in diagnosing the fatty liver. The ARFI elastography is developed to measure the velocity of the shear wave, which is related directly to tissue stiffness. Using the ARFI system, the shear wave velocity is measured by repeating push pulses and detecting pulses across the region of interest. According to the previous studies, it has been shown that the shear wave velocity in patients with fatty liver tends to be smaller than that of normal cases [30]. Although the ARFI elastography has the ability to differentiate between normal and fatty livers, the annoying problem is that the ARFI elastography is not always available in current commercial ultrasound system.

Compared to the above methods we discussed, the Nakagami parametric imaging has some advantages as follows. The Nakagami parameter is a parameter that only depends on the shape of the backscattered statistics and is not affected by the magnitude of signal. As long as the signal-to-noise ratio (SNR) is large enough to reveal whole backscattered waveforms, the Nakagami parameter can be treated as an amplitude-independent parameter and the normalization step is not necessary. A previous study has performed computer simulations to suggest that the SNR of backscattered signals should be at least higher than 11 dB to offer a satisfactory performance of the Nakagami parameter for characterizing the properties of biological tissues [31]. Moreover, the Nakagami distribution has been demonstrated to be a general model for all of the distribution conditions of ultrasound backscattering, including pre-Rayleigh, Rayleigh, and post-Rayleigh distributions. Therefore, the probability distribution of the backscatter data may be better described by the Nakagami model than by other statistical models to improve the sensitivity to detecting variations in the backscatter statistics [21]. Furthermore, the Nakagami image has a good ability to visualize local statistical properties of the liver.

According to the above discussion and the current experimental findings, the final goal of ultrasound Nakagami imaging can be oriented towards clinical *in vivo* applications. The key point to implement *in vivo* applications is using ultrasound array transducers and systems for real-time imaging. Fortunately, constructing a Nakagami image just needs a standard pulse-echo system to acquire raw backscattered signals, and thus the Nakagami imaging algorithm can be compatible to most clinical systems. In the future, the computational efficiency of Nakagami imaging algorithm may be further improved to go well with the array system, and then we can combine the B-mode and the Nakagami images to provide an integrated platform for simultaneously observing the tissue anatomy and evaluating the scatterer properties of fatty livers.

### Conclusion

For this study, we presented the measurement of ultrasound Nakagami images on fatty livers in rats and compared the Nakagami parameters with the concentrations of cholesterol and triglycerides of the liver to examine the relationship between the backscattered statistics and fatty droplet concentration. The results obtained from animal experiments show that the Nakagami parameter correlates well with the concentrations of cholesterol and triglycerides in the liver, indicating that increasing fatty droplet concentration changes the backscattered statistics. Future fatty liver diagnosis may be implemented by developing functional ultrasound imaging based on the dependency of the backscattered statistics on the fatty droplet concentrations.
